# Biomarkers and neuromodulation techniques in substance use disorders

**DOI:** 10.1186/s42234-020-0040-0

**Published:** 2020-02-17

**Authors:** Bettina Habelt, Mahnaz Arvaneh, Nadine Bernhardt, Ivan Minev

**Affiliations:** 1 Department of Psychiatry and Psychotherapy, Medical Faculty Carl Gustav Carus, Technische Universität Dresden, Dresden, Germany; 2 Department of Automatic Control and Systems Engineering, University of Sheffield, Sheffield, UK

**Keywords:** Transcranial direct current stimulation, tDCS, Deep brain stimulation, DBS, Flexible electronics, Multimodal neural interfaces, Closed-loop systems, Addiction, Substance use disorders, Neurotransmitters, Neural activity, Event-related potentials, ERP

## Abstract

Addictive disorders are a severe health concern. Conventional therapies have just moderate success and the probability of relapse after treatment remains high. Brain stimulation techniques, such as transcranial Direct Current Stimulation (tDCS) and Deep Brain Stimulation (DBS), have been shown to be effective in reducing subjectively rated substance craving. However, there are few objective and measurable parameters that reflect neural mechanisms of addictive disorders and relapse. Key electrophysiological features that characterize substance related changes in neural processing are Event-Related Potentials (ERP). These high temporal resolution measurements of brain activity are able to identify neurocognitive correlates of addictive behaviours. Moreover, ERP have shown utility as biomarkers to predict treatment outcome and relapse probability. A future direction for the treatment of addiction might include neural interfaces able to detect addiction-related neurophysiological parameters and deploy neuromodulation adapted to the identified pathological features in a closed-loop fashion. Such systems may go beyond electrical recording and stimulation to employ sensing and neuromodulation in the pharmacological domain as well as advanced signal analysis and machine learning algorithms. In this review, we describe the state-of-the-art in the treatment of addictive disorders with electrical brain stimulation and its effect on addiction-related neurophysiological markers. We discuss advanced signal processing approaches and multi-modal neural interfaces as building blocks in future bioelectronics systems for treatment of addictive disorders.

## Background

Addictive disorders represent a severe health issue and a high economic burden on society. About 31 Million people suffer from substance use disorders (SUD) worldwide (Institute for Health Metrics and Evaluation 2018). The duration of hospitalisation for addiction is on the order of several months exceeding costs of about $740 billion alone in the USA for health treatment, lost work productivity and drug-related crime (National Institute on Drug Abuse 2017). For the two most frequently consumed substances, nicotine and alcohol, the probability of relapse within the first year after treatment reaches 80–95% (Hendershot et al. 2011) revealing a lack of efficacy of conventional behavioural and pharmaceutical therapies.

So called “craving”, described as a strong desire or compulsion to consume a substance in a state of withdrawal (World Health Organisation (WHO) 2019), is the core feature underlying SUD (Sayette 2016; Tiffany and Wray 2012). Assessment of craving in humans is usually performed using questionnaires which consist of self-ratings on statements reflecting urges, desires and intent of substance consumption, anticipation of positive/negative outcome and relief from withdrawal as well as lack of control of substance consumption (e.g. Alcohol Craving Questionnaire (ACQ) (Singleton et al. 1994), Marihuana Craving Questionnaire (MCQ) (Heishman et al. 2001), Questionnaire on Smoking Urges (QSU) (Tiffany and Drobes 1991)). However, these assessments have been exposed to criticism as there is neither a consistent definition of craving nor a conclusive opinion about its validity to understand addictive behaviour and relapse (Perkins 2009; Wray et al. 2013). Nevertheless, its inclusion in the International Classification of Diseases (ICD-10, WHO 2004) and the Diagnostic and Statistical Manual of Mental Disorders (DSM-5, Hasin et al. 2013), emphasises the value of craving to predict relapse and the need to define objective parameters for its evaluation (Sayette 2016).

**Fig. 1:**
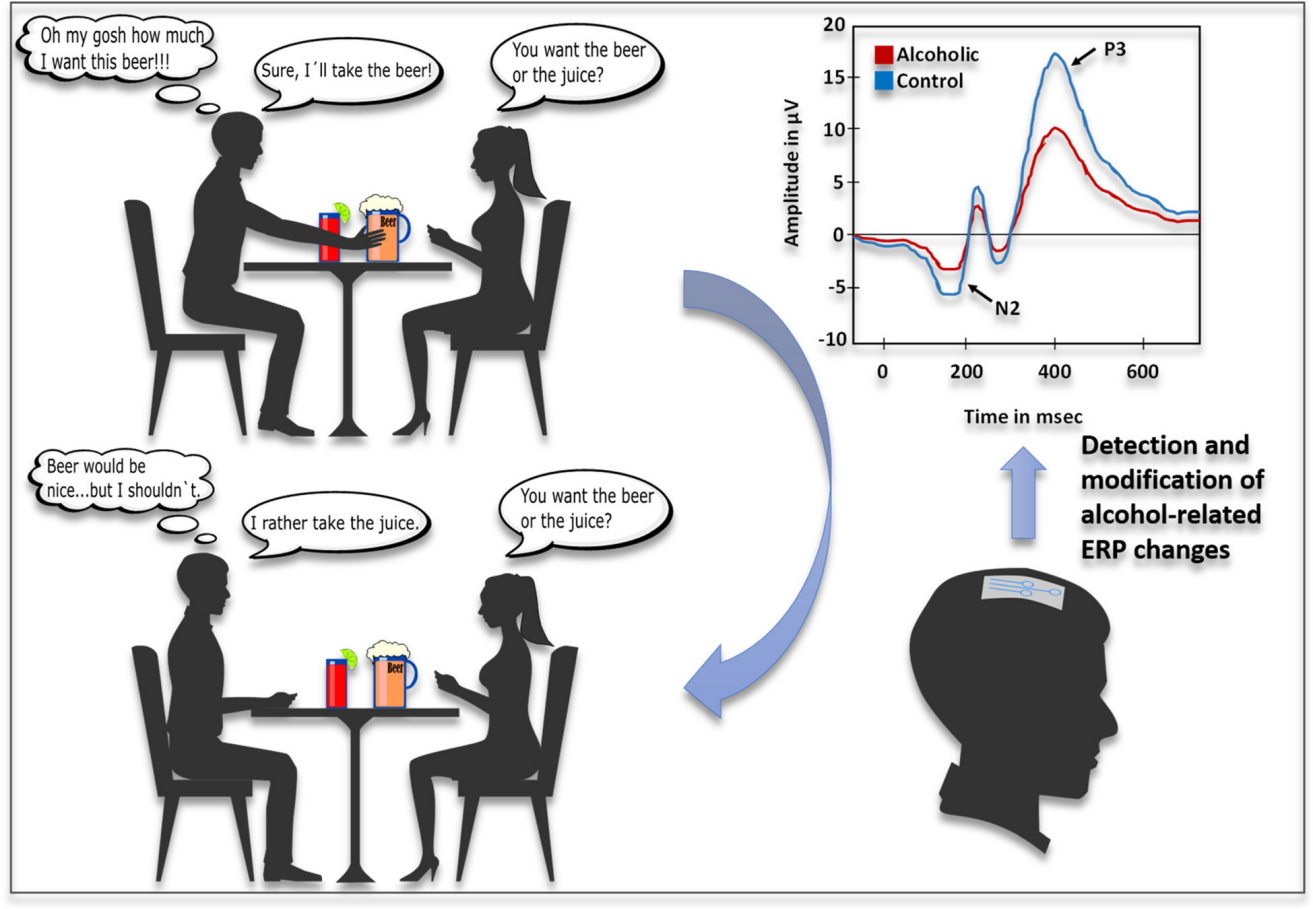
Application of neuroprosthetic devices in SUD. Drug-related stimuli can induce craving and subsequent relapse in drug addicted individuals such as a glass of beer in alcoholics. The loss of inhibitory control leading to alcohol consumption is accompanied by abnormally decreased ERP amplitudes like for N2 and P3. Neuroprosthetic systems could identify and normalise these pathological features through different brain stimulation methods leading to improved behaviour control and decreased relapse risk

Craving is particularly triggered by cues related to the substance (e.g. the sound of opening a beer bottle or the smell of a cigarette). In addicted individuals two processes are thought to consequently provoke relapse: 1.) attentional biases toward the drug-related stimulus that induces the urge to consume the drug and 2.) impaired inhibition to withstand the temptation by the drug-cue (Campanella 2016) (Fig. 1, top).

In the following review we describe neurobiological and electrophysiological parameters associated with craving behaviour in SUD. We present studies that applied brain stimulation techniques to modify these parameters within clinical treatment of addiction (Table 1). Finally, we outline the potential of intelligent bioelectronic devices in individually adapted therapeutic approaches based on neurophysiological correlates of SUD.

**Table 1:** Summary of studies investigating the effects of brain stimulation on electrophysiological correlates of addiction

Reference	Substance	Subject	Stimulation	Study design	Outcomes
Deep Brain Stimulation					
Kuhn et al. (2011)	Alcohol	human	Nacc	Flanker task	ERN↑ during 1 year of DBS
Ross et al. (2016)	Food	rat	500 msec trains, 20/130 Hz, 250–400 μA, monopolar, biphasic into CeA	Self-administration of sucrose pellets	DBS vs. sham: decreased proportion of responsive neurons to reward-related food at both frequencies
Transcranial Direct Current Stimulation					
Conti and Nakamura-Palacios (2014)	Crack-cocaine	human	20 min, 2 mA, 35 cm^2^, 1x left cathodal/right anodal vs. sham stim over DLPFC/ACC	Go/NoGo task with visual drug-related vs. neutral cues	tDCS vs. sham: N2↓ after drug-related images
Conti et al. (2014)	Crack-cocaine	human	20 min, 2 mA, 35 cm^2^, 1x per day over 5 days, left cathodal/right anodal vs. sham stim over DLPFC	Go/NoGo task with visual drug-related vs. neutral cues	1st day tDCS vs. sham: P3↑ after neutral, P3↓ after drug-related cues in DLPFC 5th day tDCS vs. sham: P3↑ after neutral and drug-related cues in DLPFC, P3↑ after drug-related cues vs. P3↓ after neutral cues in FPC, OFC, ACC
da Silva et al. (2013)	Alcohol	human	20 min, 2 mA, 35 cm^2^, 1x per week over 5 weeks anodal vs. sham stim over left DLPFC	Go/NoGo task with visual drug-related vs. neutral cues	N2↑ sham & tDCS, neutral and drug-related cues P3↑ sham, neutral cues P3↓ sham & tDCS, drug-related cues Density of activation ↓ after tDCS vs. sham in FPC, OFC, ACC, DLPFC
den Uyl et al. (2016)	Alcohol	human	15 min, 1 mA, 35 cm^2^, 1x per day over 3 days anodal over left DLPFC/cathodal over right supraorbital area vs. sham stim	Alcohol oddball task with visual drug-related vs. neutral cues	tDCS vs. sham: P3↓ after drug-related cues
Nakamura-Palacios et al. (2012)	Alcohol	human	10 min, 1 mA, 35 cm^2^, 1x anodal over left DLPFC/cathodal over right supradeltoid area vs. sham stim	passive listening to auditory drug-related vs. neutral cues	tDCS vs. sham: P3↑ after drug-related cues
Nakamura-Palacios et al. (2016)	Alcohol Crack-Cocaine	human	20 min (Crack-users) or 2 × 13 min (Alcoholics), 2 mA, 35 cm^2^, 1x per day over 5 days, left cathodal/right anodal vs. sham stim over DLPFC	Go/NoGo task with visual drug-related vs. neutral cues	Density of P3 activation ↑ after tDCS vs. sham after drug-related cues predominantly in vmPFC
Lapenta et al. (2014)	Food	human	20 min, 2 mA, 35 cm^2^, 1x left cathodal/right anodal vs. sham stim over DLPFC	Go/NoGo task with visual food-related vs. neutral cues	tDCS vs. sham: N2↓, P3↑ in NoGo condition

## Correlates (biomarkers) of addiction

### Neurobiology

In brain imaging studies an increased activation following drug-related cues has been observed in neuronal structures involved in attention, reward perception, action selection, decision making and behaviour control (George and Koob 2010), such as the dorsolateral prefrontal cortex (DLPFC), ventral striatum (VS), amygdala, orbitofrontal cortex (OFC) and anterior cingulate cortex (ACC) (Chase et al. 2011; Hayashi et al. 2013; Kühn and Gallinat 2011). Furthermore, craving is associated with changes in neurotransmitter contents within these areas as revealed by measurements using implanted biosensors in rodent models that received drug injections, self-administered drugs via lever pressing or showed drug-induced conditioned place preference (D’Souza 2015; Lenoir and Kiyatkin 2013; Malvaez et al. 2015; Rahman et al. 2005; Scofield et al. 2015; Wakabayashi and Kiyatkin 2012).

Dopamine, which is involved in various cognitive processes like decision making and action planning, plays a key role in the reinforcement of actions associated with reward and positive feelings. Repetitive drug consumption increases the activity of dopaminergic neurons elevating dopamine concentration in the ACC, amygdala and nucleus accumbens (NAcc) (Vollstädt-Klein et al. 2012; Ward et al. 2009). Particularly, in the NAcc, ventral tegmental area (VTA) and prefrontal cortex (PFC), dopamine is also co-released with glutamate, an excitatory neurotransmitter influencing impulsive behaviour and attentional, motivational and emotional processes in the context of drug-related stimuli. Drugs affect the glutamate metabolism in different ways: while cocaine intake activates dopamine D1 receptors that subsequently increase excitatory glutamate transmission, heroin and alcohol do so by reducing GABAergic interneuron inhibition on presynaptic glutamate transmission (D’Souza 2015; Lüscher and Malenka 2011).

Also increased levels of serotonin, a regulator of emotions, stress and appetite, are induced by various drugs such as alcohol, cocaine and methamphetamine. A subsequent dysregulation of the serotonin metabolism is associated with anhedonia, dysphoria, depression and anxiety during abstinence and consequently triggers drug seeking (Belmer et al. 2016; Müller et al. 2010; Ward et al. 2009).

### Electrophysiology

Electroencephalography (EEG) is a non-invasive, painless, low cost and easy-to use-method to record electrical brain activity with a high temporal resolution.

While resting state-EEG records mainly spontaneous neural activity, indicating a rather fundamental brain state (Bai et al. 2017), Event-Related Potentials (ERP) are induced by a stimulus representing associated sensory, cognitive, affective and motor processes (Kappenman and Luck 2011).

ERP are commonly defined as time-locked local positive or negative maxima within voltage waveforms recorded during EEG that arise from postsynaptic potentials (PSP) of a large amount of spatially aligned cortical pyramidal neurons. PSP are based on neurotransmitter bindings to the postsynaptic cell membrane causing opening/closing of ion channels and subsequent alterations in electrical potentials. ERP-related voltage changes are on the order of a few microvolts (μV) lasting tens to hundreds of milliseconds (msec). They are commonly named according to their polarity (positive = P, negative = N) and latency (either in milliseconds or as their order of appearance within the recorded waveform) (Kappenman and Luck 2011).

Studies to investigate ERP in the context of SUD involve visual or auditory substance-related stimuli presented commonly in inhibitory control paradigms such as oddball, Go/NoGo, Stroop or Flanker tasks (Moeller and Paulus 2018). ERP discussed in the context of SUD include the components N170, N2/mismatch negativity (MMN), N400, P50, N1/P2, P3, the late positive potential (LPP) and the error-related negativity (ERN) and are now described in more detail.

#### N170

The N170 component occurs between 130 and 200 msec after stimulus onset with largest amplitudes at occipito-temporal electrode sites. It has been shown to be most pronounced when images of faces or eyes were used as stimuli (Earp and Everett 2013). With regard to SUD, prolonged latencies and decreased amplitudes of the N170 component were detected in alcoholic individuals vs. controls in response to face images with varying emotional expressions (Maurage et al. 2007, 2008) and in multiple substance-addicted mothers when confronted with pictures of infant faces (Landi et al. 2011). These results might indicate altered visual or emotional processing in SUD and a diminished neural reaction to reward (Rutherford et al. 2013). In response to substance-related cues, alcohol-addicted individuals displayed larger NoGo N170 amplitudes and a higher rate of relapse in a 3-month follow-up assessment compared to abstinent patients suggesting that the N170 might be useful in evaluating substance-related visual cue sensitivity and treatment success (Matheus-Roth et al. 2016).

#### N2

The N2 component occurs mainly at frontal electrode sites approximately 100–350 msec after stimulus onset and reflects an automatic response to changes in stimulus properties (Sur and Sinha 2009). Its subcomponent N2a or MMN peaks approximately 150 msec post-stimulus and is usually induced by a deviant auditory cue in a series of frequent, similar sounds (Campanella et al. 2014).

In alcohol addicts vs. controls, reduced N2 amplitudes for Go as well as NoGo task conditions (Pandey et al. 2012) and even absence of the N2 component were observed in heavy drinkers, while higher N2 amplitudes for NoGo trials compared to Go trials for alcohol-related cues were detected in study participants with a high level of alcohol avoidance (Kreusch et al. 2014). A reduced N2 in NoGo task conditions has also been observed in tobacco smokers (Buzzell et al. 2014), cannabis users (Nicholls et al. 2015), and, besides a prolonged latency, also in heroin addicts (Motlagh et al. 2016, 2017), while consumers of methylenedioxymethamphetamine (Ecstasy, MDMA) displayed increased N2 amplitudes in a semantic retrieval task (Roberts et al. 2013).

Longer latencies and increased N2 amplitudes in a visual distractor task have also been observed in multiple substance-addicts, that discontinued treatment early when compared to those participants that continued treatment (Fink et al. 2016). Such data clearly illustrate the value of the MMN as a sensitive marker of impaired cognitive control and treatment success (Buzzell et al. 2014).

#### N400

The N400 is observed between 200 and 600 msec after stimulus onset predominantly at centro-parietal sites. As the N400 mostly occurs following visual or auditory meaningful words, it has been associated with language comprehension, semantic information processing and semantic memory (Kutas and Federmeier 2011). The N400 has been primarily studied in schizophrenia and correlates with deficits in interpreting associations between objects or events that underlie unusual thoughts and delusions (Jacob et al. 2019; Kiang and Gerritsen 2019). But also in the context of SUD, reduced amplitudes and increased latencies of the N400 component have been observed in alcohol addicts (Ceballos et al. 2005) and their offspring (Roopesh et al. 2009) as well as frequent cannabis consumers (Kiang et al. 2013), who additionally displayed a disturbed semantic comprehension.

#### P50

The P50 component peaks between 40 and 75 msec after an auditory stimulus mainly at central electrode sites. After the second of two identical sounds (“paired click” paradigm) it appears with a reduced amplitude reflecting an inhibited response to repetitive stimuli (Campanella et al. 2014; Sur and Sinha 2009). The P50 sensory gating works as a preattentional inhibitory filter mechanism enabling attention to salient stimuli while ignoring redundant or trivial information (Lijffijt et al. 2009; Sur and Sinha 2009). A pronounced P50 sensory gating effect has been associated with better task performance and faster reaction times and is supposed to reflect an individual’s ability to control attention and inhibition of conflicting information input (Lijffijt et al. 2009).

Deficits in P50 suppression have been observed in a variety of psychiatric diseases including SUD. Reduced P50 difference scores relative to controls in the paired-click paradigm have been detected in alcoholic individuals (Marco et al. 2005; Sklar and Nixon 2014), tobacco smokers (Brinkmeyer et al. 2011; Knott et al. 2010a, 2010b), cannabis consumers (Broyd et al. 2013, 2016), cocaine addicts (Boutros et al. 1993; Boutros et al. 2002; Fein et al. 1996) and in those under the influence of acute amphetamine application (Light et al. 1999), suggesting it is a marker of substance-related impaired early sensory processing.

#### N1/P2

The P2 occurs between 150 and 250 msec after a visual or auditory stimulus at fronto-central areas. Together with the N1 component, that peaks 80–150 ms post-stimulus at centro-temporal (auditory) or occipital (visual) areas, the P2 is also involved in sensory gating but supposedly underlies different cognitive mechanisms than the P50 related to triggering and allocation of attention (Lijffijt et al. 2009). Here, increased amplitudes of N1 and decreased amplitudes of P2 reflect the case of consciously attending to a stimulus (Crowley and Colrain 2004). Deficits of N1/P2 sensory gating revealed by decreased amplitudes of both components have been observed in cocaine addicts vs. controls with additionally prolonged latencies with comorbid paranoia (Boutros et al. 2006), suggesting that the N1/P2 complex correlates with perceptual aberrations (Gooding et al. 2013). Using a visual two-alternative forced choice task, decreased P2 amplitudes have also been detected in frequent MDMA consumers (Casco et al. 2005). Diminished auditory N1/P2 amplitudes were further detected in former and current tobacco smokers vs. never-smokers correlating with years and amount of daily consumed cigarettes (Jawinski et al. 2016).

In patients undergoing methadone maintenance treatment for opiate addiction, Wang et al. (2015) detected increased P2 amplitudes compared to healthy controls in reaction to target stimuli in an auditory oddball task suggestive of chronically altered sensory information processing following prolonged opiate consumption.

#### P3

The P3 is a large, long-lasting component observed between 300 and 700 msec at central-parietal sites after onset of visual or auditory stimuli. Its amplitude varies in dependence of frequency and significance of the stimulus and motivation and vigilance of the subject. The P3 has been associated with a wide range of attentional, memory and premotor decisional processes including activation of inhibitory mechanisms (Campanella et al. 2014). A reduction in the P3 amplitude in common oddball tasks has been shown to be a potential indicator of impaired inhibitory processes and has been suggested an endophenotype for externalizing psychopathology that should consequently be used for its diagnosis and treatment (Brennan and Baskin-Sommers 2018; Campanella 2013).

In numerous studies with alcohol use disorder (AUD) patients, ERP following alcohol-related visual or auditory stimuli displayed reduced amplitudes and increased latencies of the P3 component compared to control subjects (Cohen et al. 2002; Kathmann et al. 1996; Kreusch et al. 2014; Maurage et al. 2008). This has been associated with an impaired behaviour control and a higher relapse probability (Iacono et al. 2002; Polich 2007). Similar findings were reported for animal models of alcohol addiction (Criado and Ehlers 2009; Ehlers et al. 2014; Slawecki et al. 2003). The P3 component is therefore considered the best predictor and biomarker for the relapse risk after alcohol withdrawal (Petit et al. 2015). In addition, the P3 has been suggested as a potential marker for vulnerability to develop AUD. In alcohol naïve, young adult offspring of families with a history of AUD, altered P3 patterns with higher amplitudes compared to controls were identified. Furthermore, P3 amplitudes were equally high in Go and NoGo conditions. This leads to the assumption that similar effort might be necessary to distinguish between Go and NoGo and to inhibit behaviour in the NoGo condition (Domínguez-Centeno et al. 2018).

Similarly, a number of studies in tobacco smokers vs. non-smokers reported increased P3 amplitudes to smoking-related visual cues and decreased NoGo P3 amplitudes in Go/NoGo tasks as an indicator for conditioned learning, craving, impaired behaviour control and increased relapse risk (Littel and Franken 2012; Luijten et al. 2016; Mashhoon et al. 2018; Piasecki et al. 2017; Yin et al. 2016).

In cannabis users vs. controls, P3 amplitudes were reduced in a dose-dependent manner in a visual selective attention task (Böcker et al. 2010) and an auditory oddball task (D’Souza et al. 2012). Remarkably, the reduction in amplitudes persisted hours after consumption.

The P3 component has also been shown to display deterioration of clinical symptoms by cannabis consumption: cannabis-induced alterations in emotion processing, revealed by decreased P3 amplitudes during an emotional expression recognition task, were present in depressive patients and healthy controls but more pronounced in diagnosed depression (Troup et al. 2017). In psychotic patients cannabis-induced decreases in auditory P3 correlated with severity of psychopathology (van Tricht et al. 2013).

Disturbed cognitive processing related to psychosis as indicated by the P3 has further been investigated in the context of psychedelic drugs. Application of psilocybin to healthy subjects induced psychotic-like symptoms and a disrupted auditory P3 that decreased with increasing intensity of the psychedelic status (Bravermanová et al. 2018). In previous moderate and heavy MDMA consumers, reduced P3 amplitudes in a visual discrimination task even 6 months after last drug intake indicate long lasting impairments in cognitive performance (Casco et al. 2005). Decreased midline NoGo P3 amplitudes in a Go/NoGo task further point to disturbed inhibitory brain mechanisms in MDMA consumers (Gamma et al. 2005).

Effects of heroin on P3 are inconsistent. A reduced P3 amplitude in heroin users compared to controls was observed during an auditory oddball task (Marques-Teixeira and Barbosa 2005; Motlagh et al. 2017) and short memory task (Papageorgiou et al. 2004), while others did not detect differences (Wang et al. 2015; Yang et al. 2009) or report increased P3 amplitudes during a visual oddball task with heroin-related stimuli (Lubman et al. 2007; 2008).

Cocaine addicted individuals displayed reduced P3 amplitudes to visual (Conti et al. 2015) and auditory (Moeller et al. 2004) drug cues. Furthermore, the P3 component has been shown to be a suitable marker to predict relapse also in former cocaine consumers (Bauer 1997).

In methamphetamine consumers, increased P3 amplitudes to drug-related pictures (Shahmohammadi et al. 2016) and words (Haifeng et al. 2015) have been detected at the beginning of a therapy. With increasing duration of abstinence, P3 amplitudes normalised suggesting treatment success.

#### LPP

The Late Positive Potential (LPP) is a slow wave occurring 400–800 msec after presentation of visual stimuli at fronto-central sites. It is elicited by both, pleasant and unpleasant, emotionally salient stimuli and is therefore associated with emotional arousal (Gibney et al. 2019).

Increased LPP amplitudes and high craving scores to drug-related stimuli have been observed in cocaine-addicts suggesting that the LPP reflects stimulus approaching behaviour (Franken et al. 2008). These effects can be effectively reversed by prolonged duration of abstinence as shown in a 6-month follow up assessment. Therefore, the LPP might serve as a cocaine-related attention bias in addicted individuals and an indicator for treatment success (Parvaz et al. 2017).

In a study with tobacco smokers vs. non-smokers, smoking-related pictures induced increased LPP amplitudes in both groups (Deweese et al. 2018) pointing to the fact that positive and negative feelings towards the stimulus influence the LPP. Nevertheless, health warning stimuli on cigarette packages resulted in reduced and delayed LPP in smokers vs. non-smokers (Stothart et al. 2016).

#### ERN

The error-related negativity (ERN) is a negative, stimulus-independent component originating from the ACC and observed at fronto-central areas approx. 60–110 msec after a subject realizes they relayed a wrong (or missing) response. The ERN is considered to be a monitoring system for error detection to prevent uncontrolled, undesirable actions (Wauthia and Rossignol 2016).

Normally, adolescents show increasing ERN with age. In a longitudinal study using a Flanker task, adolescents, who consumed tobacco later in early adulthood, displayed reduced amplitudes at ages 14 and 16 and slower developmental ERN changes suggesting that an impaired error monitoring could predict development of addictive behaviour (Anokhin and Golosheykin 2015). These findings are further supported by studies with individuals having a family history of SUD and are therefore at risk also to develop an addiction: offspring of cannabis consumers (Euser et al. 2013) and alcoholics have been shown to display reduced ERN similar to already addicted individuals, supporting the ERN as an indicator of disease status (Gorka et al. 2019). Also in cocaine-dependent patients vs. controls reduced ERN amplitudes in a Flanker task have been shown to reliably predict relapse within 3-month (Marhe et al. 2013).

## Neuromodulation strategies for addictive disorders

### Deep brain stimulation

Deep Brain Stimulation (DBS) is delivered via invasive electrodes implanted in specific brain areas. Such systems are fully implanted and additionally include a subcutaneously placed pulse generator (Alonso et al. 2016). DBS for SUD has been applied to a small number of severe and otherwise treatment resistant cases. In AUD, DBS of the NAcc has been shown to successfully decrease craving and enable long-term abstinence (Heinze 2009; Kuhn et al. 2011; Müller et al. 2016; Voges et al. 2013). NAcc-DBS has also been successfully applied in a methamphetamine-addicted patient that remained abstinent for the 2-year follow up period (Ge et al. 2019). Application of simultaneous DBS of the NAcc and anterior limb of the internal capsule (ALIC) in heroin addicts resulted in decreased craving and abstinence in 5 out of 8 patients (Chen et al. 2019).

Further support for NAcc-DBS comes from rodent studies, where it has been shown to effectively reduce seeking for cocaine (Guercio et al. 2015; Hamilton et al. 2015), methamphetamine (Batra et al. 2017) and sucrose (Guercio et al. 2015) suggesting that NAcc-DBS diminishes general reward seeking behaviour.

So far, investigations on effects of DBS on ERP have been restricted to patients receiving DBS as treatment for Parkinson’s disease (PD), obsessive compulsive disorders (OCD) or treatment resistant depression. Thereby, DBS has widely been applied to the subthalamic nucleus (STN) that is involved in motor performance and also non-motor functions such as behaviour inhibition and error monitoring. Recordings of visual ERP in PD patients with vs. without bilateral DBS of the STN induced a stimulation intensity-dependent decrease of amplitudes of N70 and P1 (Jech et al. 2006). In a visual working memory task, STN-DBS furthermore reduced N2 amplitudes while increasing its latencies (Selzler et al. 2013). Using standard auditory oddball paradigms, no significant changes have been detected in amplitudes of N1, N2, P2 and P3 in PD patients when STN-DBS was switched on vs. off (Kovacs et al. 2008; Naskar et al. 2010). However, Kovacs et al. (2008) found a positive correlation between fronto-central P3 amplitudes and optimal stimulation voltage and between P3 latencies and duration of the disease. Furthermore, increased N1 latency towards the target tone was observed when DBS was turned on (Naskar et al. 2010). Gulberti et al. (2015) could restore deficits of auditory sensory gating in PD patients by normalizing abnormally increased N1/P1 amplitudes and N1 latencies by chronic STN-DBS.

OCD patients receiving bilateral STN-DBS displayed a reduced amplitude and increased latency of the P3 component in conjunction with faster reaction times during a stop-signal task when stimulation was switched on (Kibleur et al. 2016).

Kibleur et al. (2017) applied DBS to the subgenual cingulate gyrus (CG25) in patients suffering from treatment resistant depression and analysed its effect on emotional processing in a Stroop task showing pictures of faces. DBS significantly reduced overall N170 amplitudes and influenced emotional valence as revealed by larger N170 amplitudes in emotional vs. neutral control conditions. In addition, increased P3 amplitudes were observed in neutral vs. emotional trials. In a long-term study with depressive patients treated with DBS of the subcallosal cingulate (SCC), Hilimire et al. (2015) detected significantly decreased P1 and P3 amplitudes for negative words in an emotional self-referential task. This suggests that SCC-DBS changes automatic attentional focusing and controlled processing of negative information.

Beneficial effects of DBS on impaired auditory information processing and sensory gating have also been shown in a rat model of schizophrenia. By applying bilateral DBS to the ventral hippocampus, deficits of auditory P50 and N1 in the thalamic mediodorsal nucleus (MD) and infralimbic cortex were reversed (Ewing and Grace 2013).

### Electrocorticography & direct electrical stimulation

Electrocorticographic (ECoG) recordings and direct electrical stimulation (DES) of the cortex are performed with epi- or subdural electrode arrays placed on the surface of the brain. As they are not penetrating brain tissue like DBS electrodes, ECoG arrays are associated with a lower risk for side effects and a greater long-term stability (Leuthardt et al. 2006).

ECoG applications are mainly used in patients suffering from medically intractable epilepsy to localize seizure foci prior to surgical intervention (Fernández and Loddenkemper 2013) and for real-time functional brain mapping to assess language, motor performance and sensory function through application of DES via EcoG electrodes (Boyer et al. 2018; Caldwell et al. 2019; Mouthaan et al. 2016).

When used for electrophysiological measurements, ECoG–recorded ERP have been shown to correspond to ERP measured with EEG (Krusienski and Shih 2010) and might even been detected with potentially better accuracy as ECoG offers a higher spatial resolution, broader bandwidth, higher signal sensitivity and less vulnerability to artifacts compared to EEG (Leuthardt et al. 2006). This is further indicated by the application of the so called “P300 matrix speller”, an originally EEG-based BCI system that uses ERP to enable severely disabled patients to communicate. Using ECoG signals instead of EEG significantly improved and sped up spelling performance (Brunner et al. 2011; Speier et al. 2013). Miller et al. (2016) demonstrated that visual ERP and broadband changes recorded with subdural ECoG provide sufficient information to enable near-instantaneous, highly accurate identification of occurrence, timing, and category of perceived objects.

To our knowledge, ECoG and/or DES have neither been used for therapeutic applications nor for research purposes related to addictive disorders so far but have long been discussed in the context of brain computer interfaces (Caldwell et al. 2019; Kapeller et al. 2014; Leuthardt et al. 2006; Rembado et al. 2017; Schalk and Leuthardt 2011) and therefore provide a basis for medical closed-loop neuroprosthetics with a great potential also in treatment of addictive disorders.

### Transcranial direct current stimulation

Transcranial Direct Current Stimulation (tDCS) is a non-invasive, painless, inexpensive and easy-to-use brain stimulation technique with minimal side effects (Bastani and Jaberzadeh 2012). TDCS using weak electrical currents is based on a subthreshold mechanism not directly inducing pre- or postsynaptic cell firing and rather modulates spontaneous neuronal activity (Stagg and Nitsche 2011). Cathodal stimulation induces a hyperpolarisation of the resting membrane potential decreasing cortical excitability, while anodal stimulation enhances it through depolarisation of neuronal membranes (Antal et al. 2009).

In the context of addictive disorders, tDCS has been shown to successfully decrease craving scores in AUD patients (den Uyl et al. 2015; Wietschorke et al. 2016), cigarette smokers (Boggio et al. 2009), cannabis users (Boggio et al. 2010), cocaine (Batista et al. 2015) and heroin addicts (Wang et al. 2016) and methamphetamine users (Shahbabaie et al. 2014).

Several studies have been investigating the effects of tDCS on neurophysiological parameters in healthy subjects. Izzidien et al. (2016) found a significant increase in P3 power after application of anodal stimulation over the left motor cortex during a spelling task of an oddball paradigm. Keeser et al. (2011) identified increased P2 and P3 amplitudes in a working memory n-back task after anodal tDCS. Cathodal tDCS applied to the cerebellum, that supposedly interacts with cortical brain areas in attentional processing, induced an amplitude reduction of N1, N2 and P3 components for target and novel stimuli in a P3 novelty task (Mannarelli et al. 2016). Also, effects of tDCS on MMN were investigated revealing increased MMN amplitudes after anodal tDCS over the left auditory cortex and decreased sensory discrimination following cathodal stimulation in an auditory oddball paradigm (Impey et al. 2016).

## Application of brain stimulation to modulate electrophysiological correlates of addiction

There are numerous studies that investigated either effects of brain stimulation on subjectively rated craving, drug seeking and consumption in humans (reviewed e.g. in Coles et al. 2018; Luigjes et al. 2019; Salling and Martinez 2016) and animals (reviewed e.g. in Wang et al. 2018) or addressed electrophysiological correlates of addiction (reviewed e.g. in Campanella et al. 2014; Houston and Schlienz 2018; Luijten et al. 2014). Nevertheless, research on how brain stimulation modulates neurophysiological biomarkers associated with addictive behaviour remains sparse (Table 1). This is expected to be important in a closed-loop neuromodulation system where stimulation parameters will be adjusted in response to a dynamically changing biomarker.

Due to its invasiveness DBS has not yet been widely applied for the treatment of SUD. Kuhn et al. (2011) report, that after 1 year of NAcc-DBS treatment in an alcohol addicted patient, an increased, normalised ERN in parallel with decreased craving and alcohol consumption was observed. They further speculate, that such positive effect of DBS on addictive behaviour originates from an enhanced cognitive control through improved ACC functioning.

In rats, Ross et al. (2016) examined effects of DBS of the central nucleus of the amygdala (CeA) on the reward circuitry that is dysregulated in addiction. During DBS, animals stopped lever pressing for sucrose pellets and rejected freely available food rewards. Taste reactivity tests revealed aversive reactions to usually liked food tastes under influence of DBS. Neural spike recordings furthermore indicated a decreased response of CeA neurons to reward-related stimuli showing that modulation of CeA activity through DBS is able to diminish craving for rewards. To our knowledge, there are no further studies that examined the influence of DBS on substance-related ERP abnormalities.

A few more studies applied tDCS to modify addiction-related ERP changes. Conti, Nakamura-Palacios and colleagues (2014, 2016) used single and repeated bilateral tDCS in crack-cocaine addicts targeting the DLPFC and ACC. ERP measurements before and after stimulation revealed a significant reduction of the N2 over the ACC and the P3 over the DLPFC for crack-related images after a single tDCS session. Additionally, repeated tDCS up to 5 days increased the P3 component for drug-related cues over wider cortical areas, which correlated with lesser relapses and therapy dropouts and might indicate an improved behaviour control after repetitive tDCS treatment (Conti and Nakamura-Palacios 2014; Conti et al. 2014; Nakamura-Palacios et al. 2016).

In alcohol addicted individuals, repeated (two sessions) tDCS induced an increased P3 for alcohol related pictures with a dominant activation within the vmPFC (Nakamura-Palacios et al. 2016) while den Uyl et al. (2016) observed a slight reduction of the P3 component for alcohol related images after 3 stimulation sessions over the DLPFC, possibly indicating a reduced sensitivity for alcohol related stimuli. Nakamura-Palacios et al. (2012) detected an increased P3 amplitude for alcohol-related sounds after a single bilateral tDCS session. This was further accompanied with improved performance in the Frontal Assessment Battery that involves evaluation of executive functions, memory and calculation skills. As frontal dysfunction is associated with deficiencies in inhibition, tDCS-induced frontal enhancement might therefore contribute to improved behaviour control in alcoholic individuals.

Craving also plays a role in addictive eating disorders. Lapenta et al. (2014) observed a decreased N2 and enhanced P3 amplitude for visual NoGo stimuli after a single bilateral tDCS session in obese patients. Additionally, tDCS could reduce food consumption. This, furthermore, underlines the modulatory effects of tDCS on the inhibitory control circuitry.

Taken together, the presented studies demonstrate a measurable effect of tDCS on electrophysiological markers of SUD. Markers of SUD have also been shown to quantitatively correlate with the applied stimulation parameters (number of sessions, duration, dosage) and targeted brain area. The reported effects are so far restricted to the N2 and P3 components in a small number of crack-cocaine and alcohol addicted individuals. Therefore, research needs to be extended to further clarify the influence of brain stimulation on ERP in the context of substance-related disorders and to define optimal stimulation settings. For DBS the limited availability of data does not yet allow a conclusion about its influence on neurophysiological markers in SUD.

## Future directions: intelligent closed loop systems

The existing non-invasive brain stimulation studies for addictive disorders have been mainly delivered in an “open-loop”, “one-size-fits-all” fashion. In other words, the stimulation parameters (e.g. intensity, frequency, timing and target site of the stimulation) are the same for all the participants and do not vary over the time with changes in the current brain states. However, many factors including differences in brain anatomical features across participants, heterogeneity of addictive disorders and considerable changes in brain-states over time would suggest that using brain stimulation in an “open-loop”, “one-size-fits-all” fashion could not be optimum and might be the main reason for the observed large inter- and intra-individual variability in the response to brain stimulations (Li et al. 2015).

The effectiveness of brain stimulation in addictive disorders can be further enhanced by providing individualised closed-loop brain stimulation, where the parameters of stimulation (e.g. precise target site and intensity of stimulation) are defined for each individual separately and adjusted over the time based on consecutive and concurrent recordings of brain activity (Zrenner et al. 2016). For example, a pre-defined parameter (e.g. neural activity in the form of ERP) can be constantly monitored and adjusted to a desired target value. This would mean that a stimulation is applied only when an abnormal neural activity specific for a given disease (e.g. modified ERP-amplitudes and latencies in addicted individuals when confronted with drug-associated cues) is measured (Fig. 1).

Electrophysiological signals recorded immediately before starting the brain stimulation can be used to identify relatively stable spatial information that have large inter-subject but small intra-subject variability. For example, through spatial localization of certain ERP components and relevant oscillations, the precise target site and optimal position of the stimulation can be calculated for each individual using advanced high-density EEG source localization and connectivity techniques (Bergmann et al. 2016).

Recent advances in the development of machine learning algorithms in the context of EEG-based brain machine interfaces used e.g. logistic regression, Bayes estimation, support vector machines (Abibullaev and Zollanvari 2019), convolutional or recurrent neural networks (Lawhern et al. 2018; Roy et al. 2019) to identify specific electrophysiological neural features in real-time using continuously recorded neural activity. Brain potentials thereby classified as “pathological” could be used to adjust stimulation to normalise neural activity and improve behaviour control enabling an individually and situationally adapted intervention (Campanella 2013). Furthermore, this would reduce side effects observed in continuous stimulation like impaired speech, gait disorders and cognitive deficits induced by DBS (Buhmann et al. 2017).

To have a successful intelligent closed-loop brain stimulation for addictive disorders, the following methodological challenges need to be carefully addressed. The identified neural features need to be sufficiently robust against noise and artifacts in order to be reliably monitored in real-time (Arvaneh and Tanaka 2018; Kaplan et al. 2005). Moreover, the analysis pipeline needs to be sufficiently fast. Generally, the high temporal resolution provided by EEG should allow a real-time closed loop brain stimulation approach. However, the EEG is known to have a low spatial resolution (Hu et al. 2011). Thus, averaging across multiple trials might be necessary to make a reliable decision about changes of a specific EEG component. However, this might yield a delay in a closed loop system and reduces its effectiveness. Advanced machine learning algorithms and spatial filters are necessary to increase the spatial resolution of EEG signals, leading to reliable closed loop brain stimulations. Depending on the site of the stimulation, another challenge in the design of a successful closed-loop brain stimulation could be the brain stimulation-related artifacts in EEG (Helfrich et al. 2014). Removing these artifacts require advanced spatial filtering and template subtraction techniques (Marshall et al. 2016; Helfrich et al. 2014).

## Future directions: multimodal neural systems for addiction therapy

The realisation of neuroprosthetic systems for treating addictive disorders will depend on reliable monitoring of biomarkers and delivery of neuromodulation. This may be achieved using invasively implanted probes which offer advantages in terms of spatial and temporal selectivity. Electrode arrays have for some time formed the basis of clinical systems for delivering tonic stimulation to basal ganglia or for recording neural activity from the cortical surface (Coffey 2009; Cook et al. 2013). As the nervous system is also a chemical and thermal machine, recording and neuromodulation do not need to be restricted to the electrical domain only. A multi-modal approach may enable exploration of synergistic effects (decrease of stimulation/inhibition thresholds), decoupling of stimulation and recording (reducing artifacts in feedback sensor signals), side effect management or control of function in specific neural circuits (Frank et al. 2019; Kleinbart et al. 2018; Minev et al. 2015). These potential benefits are still not exploited in clinical devices but reports of hybrid interfaces are growing in the pre-clinical literature.

Neurotransmitter sensing can be facilitated by electrode arrays. These are similar to recording electrodes but sensing is enabled by electrochemical methods such as fast scan cyclic voltammetry (FSCV) or amperometry (Demuru et al. 2018). These are often made from carbon which is inert in the potential window used for sensing. In the case of dopamine sensing, FSCV is used to catalyse and detect a red-ox reaction that is specific to dopamine and occurs at low electrode potentials. Ashouri Vajari et al. (2018) have reported on the fabrication of a DBS probe for simultaneous sensing of dopamine combined with simulating electrodes. For species that are not electroactive (e.g. glutamate), the sensing electrode can be coated with a selective membrane and an enzyme (e.g. glutamate oxidase) that converts the neurotransmitter to an electroactive species (e.g. H_2_O_2_) that can be detected electrochemically (Ganesana et al. 2019). An example of an integrated sensing system for detection of dopamine, glutamate and adenosine is the wireless instantaneous neurotransmitter concentration sensing system (WINCS) which may be used as the sensing arm in closed loop or adaptive DBS (Van Gompel et al. 2010). Electrochemical methods of neurotransmitter detection have some advantages over more traditional microdialysis probes (Rogers et al. 2017) because of their smaller footprint, response speed on the order of seconds and because their fabrication shares many steps with that of traditional electrode arrays (Ou et al. 2019). Application of advanced fabrication strategies such as multi-fiber braiding and 3D printing open further possibilities for parallel detection from multiple sites and for engineering the mechanical properties of probes closer to that of soft brain tissues (Wang et al. 2019;Yang et al. 2018).

Probes that sense biopotentials in the electrical domain and deliver modulation via drugs have also attracted interest. Microfluidic chips integrated with electrodes have been used to mix and deliver up to three drugs to mouse brains and have demonstrated parallel electrical recording (Shin et al. 2015). Microfluidic conduits integrated within penetrating fiber probes have been used to deliver viral vectors for in situ optogenetic transfection. These probes also had electrodes and optrodes running parallel to the long axis of the fiber which were used to stimulate and record cells in the vicinity of the probe tip (Park et al. 2017). Minev et al. (2015) demonstrated the synergistic effect of electrical and pharmacological stimulation of the spinal cord from a surface probe, which in rats sustaining spinal cord injury was used to restore locomotion. One consideration for the deployment of microfluidics (volume flow) systems is maintaining patency of the channel in a chronically implanted setting. Disruption of the blood brain barrier, adsorption of proteins on polymer surfaces, and micromotions can lead to activation of a foreign body reaction that may interfere with the patency of the delivery system (Del Bigio 1998). Another solution may be to deliver the active molecule through a selective membrane using electrophoresis. This approach has the benefit of not requiring the transfer of large volumes of solvent to the brain and the maintenance of a patent channel. An example of this approach is a miniaturised ion pump. This has been demonstrated for the delivery of GABA, K^+^ and glutamate in rodent animal models for localised chemical neuromodulation in the brain spinal cord and cochlea (Jonsson et al. 2015; Simon et al. 2009; Uguz et al. 2017). Ionic pumps integrated with recoding electrode arrays have also been demonstrated for both in vitro and in vivo biointerfacing (Jonsson et al. 2016; Proctor et al. 2018). A drawback for this approach is the size limit of molecules that can be delivered through the membrane and such probes still require microfluidics to bring the drug close to the membrane.

A conceptual design of a multi-modal and closed-loop neuroprosthetic system for treating addictive disorders may comprise of a sensing arm to detect ERP and an effector arm to deploy neurotransmitter release, both targeting the mPFC as this area plays a superior role in craving and addictive behaviour (George and Koob 2010, 2013). In terms of probe technology we aim to fabricate soft ECoG arrays that conform to the curvature of the brain and incorporate a microfluidic channel for drug delivery. This can initially be implemented in a rat model of cue-induced reinstatement of alcohol seeking. Our ECoG arrays will thereby monitor neurophysiological parameter changes underlying the development of craving and addiction during conditioning for alcohol and modulate them afterwards with the aim of preventing relapse. Following our recent efforts to adapt 3D printing technologies for the production of fibers with electrical, optical and microfluidic functionality, we will print soft and customised implants adapted to deliver multi-modal brain interface in the rat cortex (Athanasiadis et al. 2019).

It should be noted that chemical and electrical sensing/stimulation are just two examples from a rapidly expanding toolbox of interfacing techniques. Implanted probes delivering focal cooling or heating have been demonstrated to decrease or respectively increase the excitability of surrounding cortical structures (Chen et al. 2015; Fujioka et al. 2010). Focused ultrasound can be delivered non-invasively via wearable probes and has been shown to modulate neural activity in basal ganglia in mice (Zhou et al. 2019). Optogenetic stimulation without penetrating optical probes has also been recently made possible using nanoparticle mediated upconversion of infrared light (Chen et al. 2018). Thus, it is likely that future technologies for brain-machine interfaces will either be non-invasive or will blend seamlessly with host tissues of the nervous system.

## Conclusions

The neurobiological and electrophysiological parameters described here link addiction-related behavioural deficits to particular brain regions and cognitive origins. These parameters may, in the future, provide a basis for a comprehensive diagnosis of addictive pathologies using neural interfaces. Brain stimulation methods, like tDCS, have already revealed some beneficial effects on ERP, though further research needs to be done to find the optimal treatment strategies. Multimodal neural interfaces may allow for refinement of the therapy beyond what electrical stimulation or systemic drug application can achieve in isolation. They could further enable sensing and identification of pathogenic features characteristic of individual patients and situations and could therefore open doors to the development of personalized, targeted therapies.

## Data Availability

Not applicable.

## Abbreviations

ACC: Anterior cingulate cortex
ACQ: Alcohol Craving Questionnaire
ALIC: Anterior limb of the internal capsule
AUD: Alcohol use disorder
CeA: Central nucleus of the amygdala
CG: Cingulate gyrus
DBS: Deep Brain Stimulation
DES: Direct electrical stimulation
DLPFC: Dorsolateral prefrontal cortex
ECoG: Electrocorticography
EEG: Electroencephalography
ERN: Error-related negativity
ERP: Event-related potentials
FSCV: Fast scan cyclic voltammetry
GABA: Gamma aminobutyric acid
LFP: Local field potentials
LPP: Late positive potential
MCQ: Marihuana Craving Questionnaire
MDMA: 3,4-Methylendioxy-N-methylamphetamine
MMN: Mismatch negativity
NAcc: Nucleus accumbens
OCD: Obsessive compulsive disorder
OFC: Orbitofrontal cortex
PD: Parkinson’s disease
PFC: Prefrontal cortex
PSP: Postsynaptic potentials
QSU: Questionnaire on Smoking Urges
SCC: Subcallosal cingulate
STN: Subthalamic nucleus
SUD: Substance use disorders
tDCS: Transcranial Direct Current Stimulation
vmPFC: Ventromedial prefrontal cortex
VS: Ventral striatum
VTA: Ventral tegmental area
WINCS: Wireless instantaneous neurotransmitter concentration sensing
