# Correction to: Biomarkers and neuromodulation techniques in substance use disorders

**DOI:** 10.1186/s42234-020-00043-7

**Published:** 2020-03-19

**Authors:** Bettina Habelt, Mahnaz Arvaneh, Nadine Bernhardt, Ivan Minev

**Affiliations:** 1 Department of Psychiatry and Psychotherapy, Medical Faculty Carl Gustav Carus, Technische Universität Dresden, Dresden, Germany; 2 Department of Automatic Control and Systems Engineering, University of Sheffield, Sheffield, UK

**Correction to: Bioelectron Med (2020) 6:4**

**https://doi.org/10.1186/s42234-020-0040-0**

The original version of this article (Habelt et al. 2020), published on 17 February 2020, contained incorrect sentence. In this Correction the affected part of the article is shown.

1. Incorrect sentence;

"(...) Lapenta et al. (2014) observed a decreased N2 and enhanced P3 amplitude for visual NoGo stimuli after a single bilateral tDCS session in obese patients."

2. Correct sentence;

"(...) Lapenta et al. (2014) observed a decreased N2 and enhanced P3 amplitude for visual NoGo stimuli after a single bilateral tDCS session in normal weight subjects."
